# Modulation of the thalamus by microburst vagus nerve stimulation: a feasibility study protocol

**DOI:** 10.3389/fneur.2023.1169161

**Published:** 2023-06-13

**Authors:** Ryan Verner, Jerzy P. Szaflarski, Jane B. Allendorfer, Kristl Vonck, Gaia Giannicola, Microburst Study Group, Danielle McDermott

**Affiliations:** ^1^Clinical and Medical Affairs, LivaNova PLC (or a subsidiary), London, United Kingdom; ^2^Department of Neurology, University of Alabama at Birmingham Heersink School of Medicine, Birmingham, AL, United States; ^3^Department of Neurology, 4Brain, Ghent University Hospital, Ghent, Belgium

**Keywords:** vagus nerve stimulation, drug-resistant epilepsy, focal epilepsy, generalized epilepsy, feasibility study

## Abstract

Vagus nerve stimulation (VNS) was the first device-based therapy for epilepsy, having launched in 1994 in Europe and 1997 in the United States. Since then, significant advances in the understanding of the mechanism of action of VNS and the central neurocircuitry that VNS modulates have impacted how the therapy is practically implemented. However, there has been little change to VNS stimulation parameters since the late 1990s. Short bursts of high frequency stimulation have been of increasing interest to other neuromodulation targets e.g., the spine, and these high frequency bursts elicit unique effects in the central nervous system, especially when applied to the vagus nerve. In the current study, we describe a protocol design that is aimed to assess the impact of high frequency bursts of stimulation, called “Microburst VNS”, in subjects with refractory focal and generalized epilepsies treated with this novel stimulation pattern in addition to standard anti-seizure medications. This protocol also employed an investigational, fMRI-guided titration protocol that permits personalized dosing of Microburst VNS among the treated population depending on the thalamic blood-oxygen-level-dependent signal. The study was registered on clinicaltrials.gov (NCT03446664). The first subject was enrolled in 2018 and the final results are expected in 2023.

## 1. Introduction

Device-based therapies for epilepsy aim to leverage intrinsic circuits to either interrupt or suppress epileptic activity. Two invasive, cranial procedures exist that are currently approved as adjunctive therapies to lessen the frequency of seizures in patients in whom multiple trials of anti-seizure medications (ASMs) have failed: responsive neurostimulation (RNS) and deep-brain stimulation (DBS). While DBS is an open loop device approved for the treatment of focal onset epilepsy with anterior nucleus of the thalamus as the therapy target (1), some researchers have implanted the stimulation electrodes in other thalamic nuclei e.g., centro-median nucleus (2). RNS, a closed-loop system, is currently FDA-approved for the treatment of focal onset epilepsy (3). However, similar to DBS, this system has also been implanted in patients with generalized epilepsies, including idiopathic generalized epilepsies (4) and Lennox-Gastaut Syndrome (LGS) (5), with specific trials for both indications registered with clinicaltrials.gov. Both approaches directly target brain structures with electrical energy. These approaches have been demonstrated to reduce seizure frequency by over 50% in more than 40% of patients in the first year of therapy with additional improvements observed over time. However, they carry the risk of rare but potentially severe adverse events due to the invasiveness of the implantation procedure (1, 3, 6, 7). VNS is considered a less invasive, peripheral approach to change epileptic networks, and it has been previously demonstrated to modulate epilepsy-associated brain structures (8).

The first VNS Therapy™ System received approval for the adjunctive treatment of medically refractory epilepsy in 1994 in Europe and in 1997 in the United States and consists of an implantable pulse generator (IPG) that supplies intermittent electrical stimulation to the left vagus nerve. The specific mechanism of action by which the VNS Therapy reduces seizure frequency is not precisely understood, because the physiological effects of VNS are documented as multifaceted (8). Modulation of vagus nerve firing rates has been shown to subsequently modulate central nervous system activity with this central modulation being required for the anti-seizure effect of VNS in epilepsy (9, 10).

**Figure 1:**
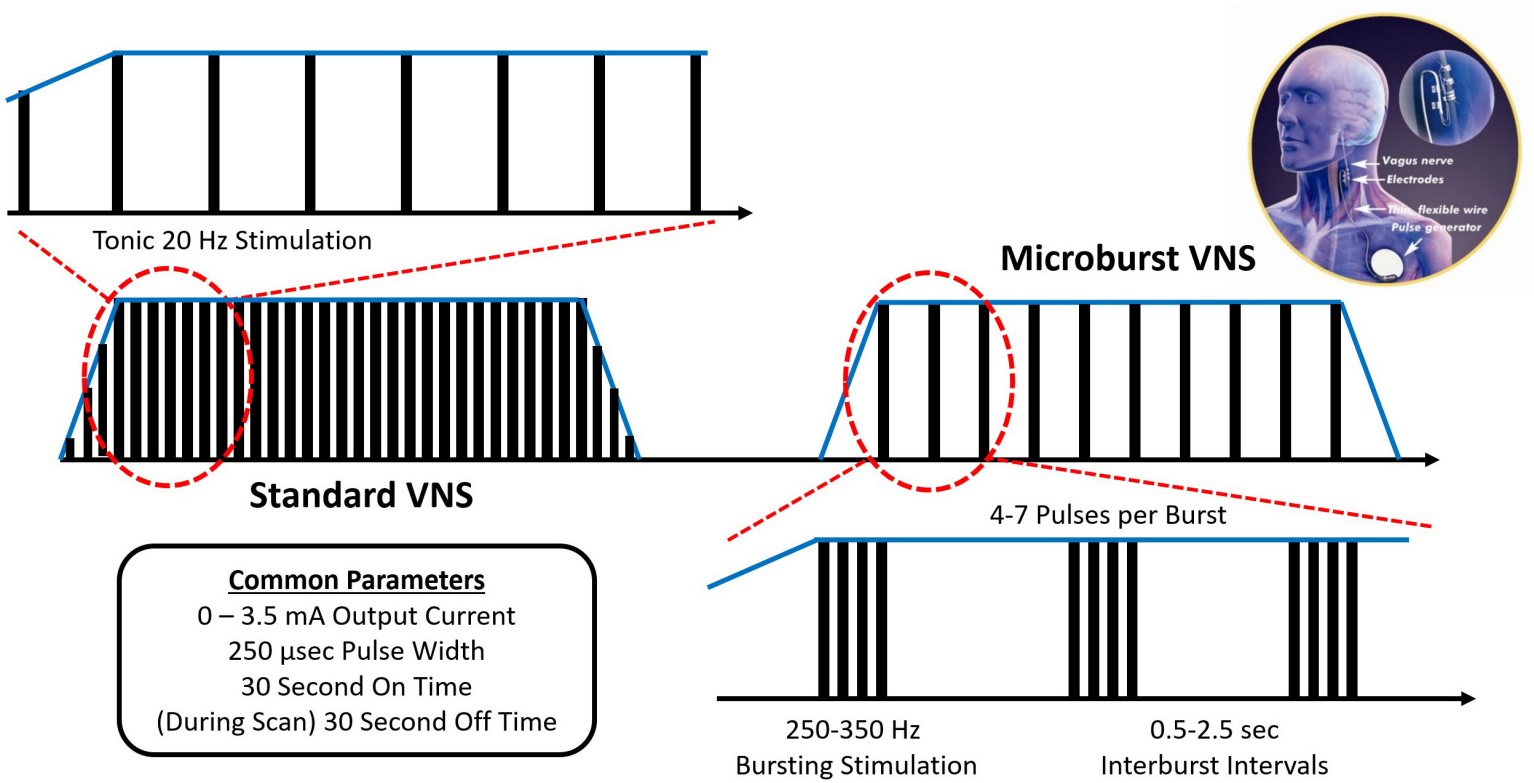
Microburst VNS consists of short bursts of pulses separated by brief off-times called interburst intervals (IBIs). The μVNS waveform incorporates 7 stimulation parameters with a range of available settings (Table 3). On compatible pulse generators, μVNS can be selected as a stimulation setting for the normal mode, the magnet mode, or the autostim mode, which can be set to different levels to deliver VNS (traditional or μVNS) at a regular cadence or based on a specific triggering event. In its current embodiment, μVNS can be delivered from standard VNS Therapy leads and implantable pulse generators with form factors similar to existing VNS devices.

In the early 2000s, an experimental VNS stimulation paradigm was developed that consists of high-frequency bursts of stimulation, herein called “Microburst VNS” (μVNS) (Figure 1). While the mechanism of traditional VNS was believed to be mediated by the nuclei closer to the brainstem, such as the nucleus of the tractus solitarius (NTS) and the nucleus of the locus coeruleus (LC), existing evidence suggests that μVNS can be employed to modulate other brain areas, including the thalamus (11, 12).

High frequency burst VNS, eventually labeled “Microburst” VNS, was first examined in primates in the early 2000s (11, 12). This stimulation protocol is similar to the one implemented in transcranial magnetic stimulation called intermittent theta burst stimulation (iTBS) that is known to affect long-term potentiation and induce cortical plasticity (13, 14). In the original experiments, standard VNS and high frequency bursts of VNS were used to evoke responses in the parafascicular nucleus of the thalamus, measured by simultaneous electrophysiological recordings. Only paired pulses of 1.5 mA at 400 μs (~5x the threshold charge density), delivered at 300 Hz, elicited a vagal evoked potential in the parafascicular nucleus that had not been previously detected in the mapping studies of vagal evoked potentials (11). Ito and Craig advocated that this effect could be due to paired pulse excitation or inhibition mechanics in the ascending vagus nerve circuits. Following this discovery, the experiment was replicated with multiunit discharges recorded in the parafascicular nucleus and the basal ventromedial nucleus (12). A series of studies in beagles and rats followed the initial primate work and investigated the impact of μVNS on imaging and biochemical markers in experimental epilepsy models (15–17). In beagles, standard VNS parameters were not associated with cerebral blood flow alterations, while μVNS caused significant hypoperfusion of the left frontal lobe and the right parietal lobe. Moreover, both standard VNS and μVNS were associated with a significant increase of norepinephrine release, suggesting evoked activation of the coeruleo-fugal pathways (15, 16). In rodents, both standard and μVNS increased the electrographic seizure threshold of pentylenetetrazole-kindled seizures, but decreased stimulus intensity may have contributed to microbursts not reaching a level of statistical significance (17). Most recently, and concurrently with the clinical feasibility study described herein, μVNS has returned to primate study in a naturally occurring model of genetic generalized epilepsy in baboons. In these animals, μVNS reduced the frequency of generalized tonic-clonic seizures except when the baboons received output currents of 0.25 mA for extended periods, suggesting a dose-response relationship (18). Baboons tolerated μVNS well, and this approach was not associated with cardiac or behavioral changes. However, transient regular muscle contractions could be detected during VNS on-times consistent with the 0.5-s interburst intervals that were not noted during wakefulness (18).

Vagus nerve stimulation is an MRI-conditional product, meaning that the collection of MRI images in patients with VNS therapy is safe provided certain use limitations are followed. One such restriction is the deactivation of the VNS device prior to introducing an implanted patient into an area of a strong magnetic field. The primary rationale for this particular restriction is the activation of a magnetically-sensitive component in the pulse generator, which could respond to the MRI's magnetic fields in a variety of ways.

However, investigators have demonstrated that it is possible to record the Blood Oxygen Level Dependent (BOLD) response of different brain regions to VNS, with the device being active during scanning. After the first demonstration of an MRI-compatible positioning of the device that avoids deactivation while the patient lies supine within the scanner (19) (only suitable for devices without “Magnet Mode”), an investigative team examined VNS-evoked BOLD responses in subjects receiving investigational VNS as therapy for treatment-resistant depression. In initial feasibility work, the team demonstrated that the phase lag from the onset of stimulation to the onset of the hemodynamic response was variable for each affected brain region but tended to be ~ 4–7 s with a similarly variable washout time of 15–25 s (20). The VNS-evoked BOLD response was dose dependent, with lower VNS charge densities resulting in significantly weaker BOLD responses (21, 22). BOLD response was diffuse and not always consistent between patients, but the most common areas of BOLD response were the thalamus, amygdala and insular cortex (20–23).

Following the preclinical history of microburst VNS investigations, and with some understanding of its mechanism of action and how that mechanism can be objectively studied, we designed a prospective, open-label, multicenter phase I clinical trial to investigate the potential risk-benefit profile of μVNS in humans. The study, registered as NCT03446664, examined over 12 months two cohorts of treatment-resistant epilepsy patients with focal-onset (including those with progression to bilateral tonic-clonic seizures) or primary (idiopathic/genetic) generalized-onset tonic-clonic seizures (PGTC). In addition to traditional outcome measures of epilepsy studies, an investigational fMRI protocol was executed in all subjects to offer personalized titration and measure the impact of μVNS on the thalamus.

## 2. Methods and analysis

**Figure 2:**
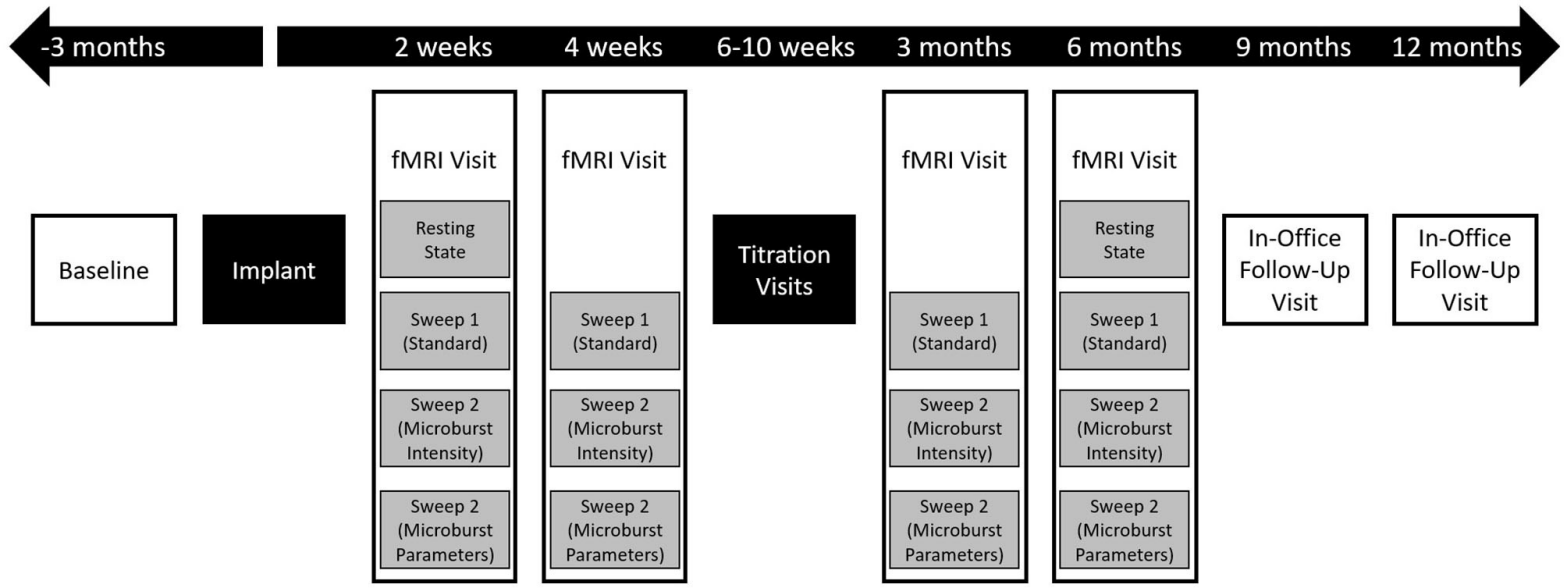
Flow chart of overall study events. After screening and consent, enrolled patients underwent a 3-month prospective baseline period followed by implantation. All subjects were then followed for up to 12 months, with the first 6 months including an intensive, imaging-guided titration program and the last 6 months including telephone and in-office visits. Clinical outcome measures were collected at all boxes shown in white, though reports of adverse events could be collected at any time, including outside of study visits. During fMRI visits (Figure 3), study outcome measures were collected sporadically during rest periods between scans to minimize the impact of the duration of the visit on the subject's schedule.

This prospective, non-randomized, interventional, open-label phase I clinical trial was designed to collect data on up to 40 subjects (20 PGTC and 20 focal onset) implanted with an investigational μVNS delivering therapy over 12 months of follow-up (Figure 2). The study was registered on clinicaltrials.gov (NCT03446664) and approved by the Institutional Review Boards and Ethics Committees of all study sites. All research procedures were conducted in accordance with the ethical principles of informed consent and the Declaration of Helsinki. All participants received care in academic hospitals from epileptologists trained in the use of the VNS Therapy System (Table 1).

**Table 1:** Microburst study sites, site investigators, and date of site authorized to start recruitment.

**Site**	**Site investigator**	**Date of site initiation**
University of Colorado—Denver	Cornelia Drees Mesha Gay-Brown Danielle McDermott Lesley Kaye	05 NOV 2018
Rush University Medical Center	Rebecca O'Dwyer	27 FEB 2018
Northwestern University	Michael Macken	07 JUN 2018
Duke University	Muhammad Zafar	25 APR 2019
Mayo Clinic Florida	William Tatum	02 JUL 2019
University of Alabama at Birmingham	Zeenat Jaisani	16 JAN 2019
University of Ghent Hospital	Kristl Vonck	04 JUN 2019
Weill Cornell Medical College	Pegah Afra	17 DEC 2018
University of Utah Health Science	Blake Newman	23 AUG 2018

### 2.1. Patient selection

Patients were recruited into a cohort based on their seizure history, baseline characteristics, and satisfaction of the inclusion criteria without meeting any of the exclusion criteria. No specific methods for patient recruitment were employed, and each site investigator was responsible for identifying appropriate patients in their practice to screen for the study. Patients recruited in the focal-onset seizure cohort had to have a clinical diagnosis of medically refractory epilepsy with focal-onset seizures, which could include seizures that secondarily progressed to bilateral tonic-clonic seizures, and had to have an average of at least three countable seizures per month during the 3-month baseline period without any seizure-free interval >30 days during the baseline period. Patients recruited into the PGTC seizure cohort had to have a clinical diagnosis of medically refractory idiopathic/genetic generalized epilepsy with generalized-onset tonic-clonic seizures, though they may also have other seizure types, and must have at least three countable seizures during the 3-month baseline period. Clinical diagnosis of PGTC seizures was required to be confirmed by historical EEG within the past 3 years by the investigator. If no historical EEG was available, a prospective EEG could be collected to verify the diagnosis by independent review.

Other inclusion criteria and exclusion criteria are described in Table 2.

**Table 2:** Additional inclusion and exclusion criteria for the Microburst Feasibility Study.

**Inclusion criteria**	**Exclusion criteria**
1) Must be on adjunctive anti-seizure medications. 2) Willing and capable to undergo multiple evaluations with fMRI, EEG, and ECG. 3) 12 years of age or older. 4) Male or non-pregnant female adequately protected from conception. Females of childbearing potential must use an acceptable method of birth control. 5) Provide written informed consent-assent/Health Insurance Portability and Accountability Act (HIPAA) authorization and self-reported measures with minimal assistance as determined by the investigator.	1) Currently using, or are expected to use, short-wave diathermy, microwave diathermy, or therapeutic ultrasound diathermy. 2) A VNS Therapy System implant would (in the investigator's judgement) pose an unacceptable surgical or medical risk for the subject. 3) A planned procedure that is contraindicated for VNS Therapy. 4) A history of implantation of the VNS Therapy system. 5) Currently receiving treatment from an active implantable medical device. 6) Presence of contraindications to MRI per the MRI subject screening record. 7) Known clinically meaningful cardiovascular arrhythmias currently being managed by devices or treatments that interfere with normal intrinsic heart rate responses (e.g., pacemaker dependency, implantable defibrillator, beta adrenergic blocker medications). 8) History of chronotropic incompetence (commonly seen in subjects with sustained bradycardia). 9) Any cognitive or psychiatric deficit found in the investigator's judgement that would interfere with the subject's ability to accurately complete study assessments. 10) History of status epilepticus within 1 year of study enrollment. 11) Dependent on alcohol or narcotic drugs as defined by DSM IV-TR within the past 2 years, based on history. Tests for drug or alcohol use will not be administered. 12) Currently being treated with prescribed medication that contains cannabis or cannabis-related substances, including recreational use. 13) Any history of psychogenic non-epileptic seizures. 14) Currently participating in another clinical study without the written approval of LivaNova.

### 2.2. Intervention

The VNS Therapy System is approved for use in epilepsy as an adjunctive treatment in reducing seizure frequency for adults and children 4 years of age or older (in Europe, all ages) with drug-resistant focal epilepsy (in Europe, also generalized epilepsy). The principal components of the system are an implantable VNS Therapy generator, a lead, and an external programming system used to change stimulation settings. The pulse generator is housed in a hermetically sealed titanium case and is powered by a single battery. Electrical signals are transmitted to the left cervical vagus nerve through the lead. The system is manufactured by LivaNova USA, Inc.

Subjects enrolled in this study received the investigational M1000C μB SenTiva™ VNS Therapy System along with a commercial, FDA approved VNS Therapy System lead, either the M302, M303, or M304. The M1000C unit was programmed to provide investigational “microburst” stimulation patterns (Figure 1). Microburst stimulation consists of short bursts of pulses separated by brief off-times called “interburst intervals” (IBI) (13). The microburst waveform can be fully described by 6 stimulation parameters with a range of available settings: Output Current, Pulse Width, Signal Frequency, Duty Cycle, Interburst Interval, and Number of Pulses (Table 3). The M1000C investigational VNS Therapy system provided all the basic functionality of previous VNS Therapy models as well as the new microburst feature under investigation.

**Table 3:** VNS settings, microburst and standard VNS on the M3000C investigational VNS programming system.

	**Output current (mA)**	**Pulse width (μsec)**	**Signal on time (sec)**	**Signal off time (min)**	**Signal frequency (Hz)**	**Interburst interval (sec)**	**Number of pulses**
Standard VNS	0–2 in 0.125 mA increments; 2–3.5 in 0.25 mA increments	100, 130, 150, 200, 250, 300, 350, 400, 450, 500	7, 14, 21, 30, 60	0.2, 0.3, 0.5, 0.8, 1.1, 1.8, 3, 5–55 (by 5), 60–180 (by 30)	1, 2, 5–30 in 5 Hz increments	N/A	N/A
Microburst VNS	0–2 in 0.125 mA increments; 2–3.5 in 0.25 mA increments	100, 130, 150, 200, 250, 300, 350, 400, 450, 500	7, 14, 21, 30, 60	0.2, 0.3, 0.5, 0.8, 1.1, 1.8, 3, 5–55 (by 5), 60–180 (by 30)	100–350 in 50 Hz increments	0.05, 0.1, 0.2, 0.3, 0.4, 0.5, 1, 1.5, 2, 2.5, 3, 4, 5, 6	2, 3, 4, 5, 6, 7

In addition to the μVNS settings, the M1000C VNS Therapy System includes a “parameter sweep” feature that is designed to allow for the stimulation of the vagus nerve using up to 7 sets of existing parameter values (e.g., the choice of a value for each VNS parameter creates one set) over a short period of time. The parameter sets were delivered sequentially at pre-defined intervals (e.g., 5 min of parameter set 1, 5 min of parameter set 2, and so on). Simultaneously, the parameter sweep feature disengaged the functionality of the reed switch, which is an electrical component that responds to the presence of a strong magnetic field by opening or closing a circuit. Disengaging the reed switch allowed the M1000C VNS Therapy system to deliver stimulation inside the bore of a MRI scanner for investigational purposes.

All patients received an active VNS implant. There was no group with inactive or intentionally low output μVNS.

### 2.3. Titration strategy

**Figure 3:**
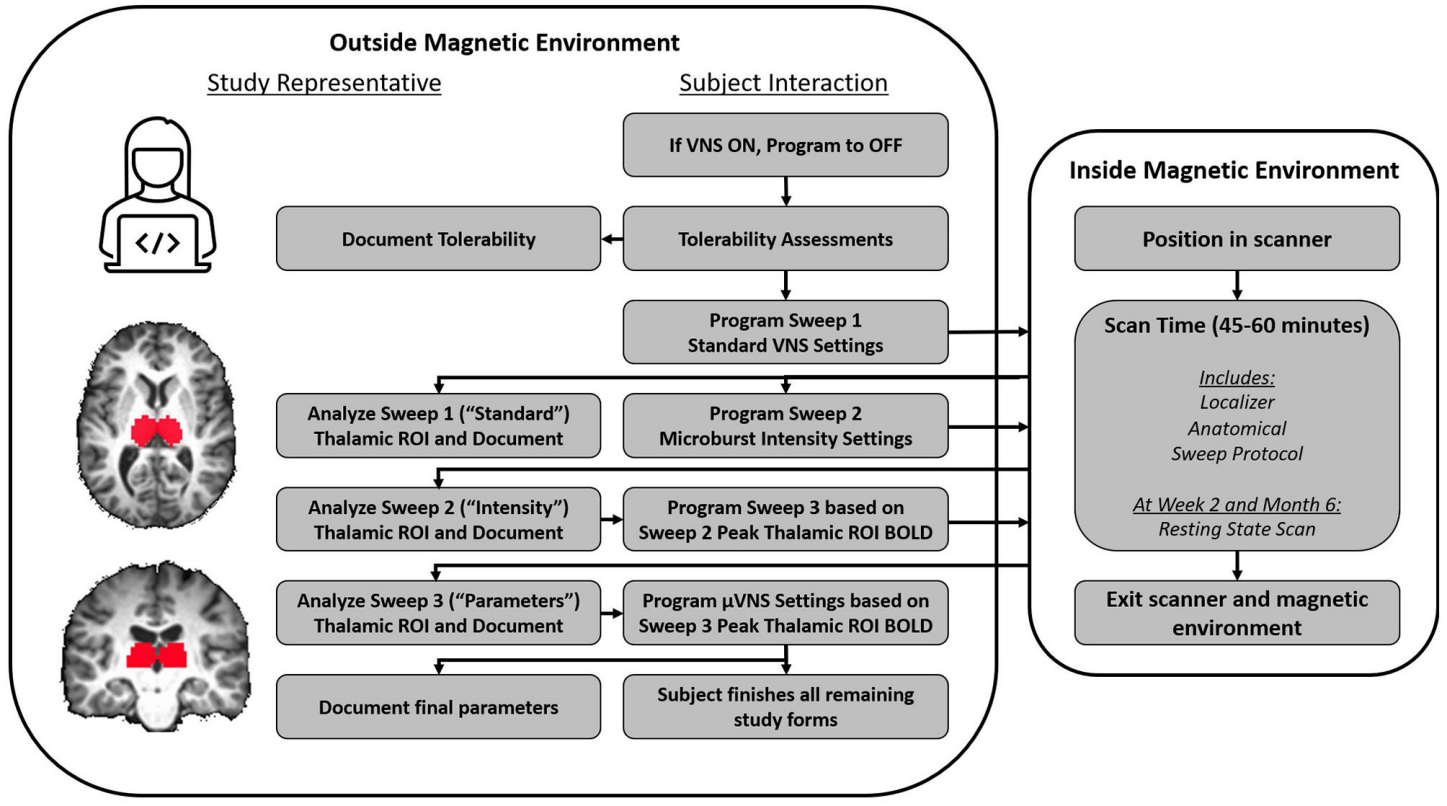
Flow chart of activities during an fMRI titration visit. The device was initially deactivated, and then, a tolerability protocol was followed. The purpose of the tolerability protocol was to identify VNS and μVNS intensities that evoked intolerable side effects so that side effects that induced involuntary movement (e.g., cough) could be avoided in the scanner. Based on the tolerability assessments, a maximum intensity was determined for VNS (Sweep 1) and μVNS (Sweeps 2 and 3). A study representative then programmed the Sweep 1 parameters into the device's “Parameter Sweep Mode”, which is an investigational function that allows the pulse generator to sequentially modify programming settings at a future time (e.g., in the scanner). The subject was then positioned into the scanner, and multiple functional and anatomical scans were collected. When Sweep 1 concluded, the subject was removed from the scanner and study representatives programmed Sweep 2 based on the tolerability assessment and re-admitted the subject to the scanning environment. While Sweep 2 was underway, study representatives analyzed the Sweep 1 fMRI data to identify the settings associated with the peak BOLD response in the thalamic ROI. When Sweep 2 concluded and the subject was removed from the scanner, study representatives analyzed the Sweep 2 fMRI results similar to the procedure for the Sweep 1 results; however, when programming the subject for Sweep 3, the optimal μVNS intensity identified in the Sweep 2 results was used. When Sweep 3 concluded, a final analysis was completed by study representatives to identify the μVNS parameters that the subject would leave the clinic with.

A critically important element to the design of this study was the fMRI-guided titration strategy (Figure 3). Post-implant, at weeks 2, 4, 12, and 24, patients were required to return to their study site for a follow-up visit that included a personalized, BOLD-driven titration protocol. Subjects proceeded directly to an MRI scanning facility at the hospital for these visits, where they met with the site investigator, a sponsor's clinical engineer, and other MR facility personnel. First, tolerability of both standard VNS and μVNS was assessed to determine the maximum tolerability output current and pulse width for that study visit. Following the maximum tolerability determination, the subject's device was programmed using the parameter sweep function to deliver up to seven unique parameter sets over the course of the following 45–60 min (see Tables 4A, B for examples). The subject was then placed into the MRI scanner while the parameter sweep function was active. Each patient underwent a series of three fMRI scans with parameter sweeps per visit, totaling 45–60 min per scan or up to 180 min of scanning per visit day.

**Table 4A:** Programming table for parameter Sweep 2, using an exemplar tolerability value of 1 mA.

**Step**	**Output**	**PW (μsec)**	**SF (Hz)**	**IBI (s)**	**Pulses**	**ON (s)**	**OFF (min)**
1	0 mA	VNS off. Step 1 used to position subject in scanner.					
2	0.375 mA	250	300	2.5	7	30	0.5
3	0.500 mA	250	300	2.5	7	30	0.5
4	0.625 mA	250	300	2.5	7	30	0.5
5	0.75 mA	250	300	2.5	7	30	0.5
6	0.875 mA	250	300	2.5	7	30	0.5
7	1.00 mA	250	300	2.5	7	30	0.5

**Table 4B:** Programming table for parameter Sweep 3.

**Step**	**Output**	**PW (μsec)**	**SF (Hz)**	**IBI (sec)**	**Pulses**	**ON (sec)**	**OFF (min)**
1	0 mA	VNS off. Step 1 used to position subject in scanner.					
2	Optimal intensity setting selected from Sweep 2 analysis.	250	250	1.5	4	30	0.5
3		250	300	1.5	4	30	0.5
4		250	300	0.5	4	30	0.5
5		250	300	0.5	7	30	0.5
6		250	300	2.5	7	30	0.5
7		250	350	2.5	7	30	0.5

The principal objective of each 45–60 min scan was to examine the BOLD signal within a region of interest (ROI) centered over the left and right thalamus. After completion of the structural scanning protocol (structural voxel size not larger than 1 mm^3^ isotropic), a 30-min fMRI sequence was initiated at the same time as the first set of pre-programmed parameters from the parameter sweep (functional voxel size not larger than 4 mm^3^ isotropic). The parameter sweep programmed VNS settings in a 30 s ON/30 s OFF manner and switched to a new group of VNS settings every 5 min. This paradigm permitted a later off-line analysis of BOLD signal in the ON vs. OFF state for each pre-programmed group of settings. Maximal BOLD signal increases from each 45–60 min MR session within the thalamic ROI were used to identify settings for the next parameter sweep, refining the VNS programming with each scanning session. Settings for the next scan were determined by identifying settings in the preceding scan that resulted in the greatest thalamic ROI BOLD intensity and by the maximum *t*-value calculated from at least 2 contiguous voxels.

The first scan of a visit day assessed standard VNS settings, and the output current was the only parameter that varied during the fMRI session. Subjects started with a resting scan with no stimulation (VNS inactive) and then proceeded from a low-intensity stimulation to a higher intensity stimulation, as determined by the tolerability assessment from that visit day. After the scan, the subject was given a short break while the sponsor's engineer analyzed the fMRI data using a customized processing pipeline utilizing the Analysis of Functional NeuroImages (AFNI) software (24) to determine the output current associated with peak thalamic ROI BOLD signal increase. The subject was then programmed for a μVNS sweep that also assessed output current, starting from low intensities and moving to higher intensities limited by the tolerability. Patient tolerance to stimulation was assessed separately for both standard VNS and μVNS, so the output current settings were not always the same between the first and second parameter sweep protocols. After the second scan, the subject again exited the scanner and the sponsor's engineer analyzed the data. For the third scan, the patient's parameter sweep was programmed to the output current intensity of the thalamic ROI BOLD peak from the second scan. At that intensity, the other μVNS settings of IBI, number of pulses, and signal frequency were adjusted. After this scan was completed, the sponsor's engineer again analyzed the fMRI data using the custom fMRI processing pipeline. The pulse generator was programmed to the intensity (output current and pulse width) resulting from the second scan and the μVNS settings that drove peak thalamic ROI activation in the third scan. The patient left the clinic with these settings.

After the 4-week MRI visit, patients were asked to visit the clinic once every 2 weeks for titration of their output current, up to the 12-week MRI visit. While the relationship between standard VNS titration and μVNS titration is not fully understood, interim titration visits to adjust output current were performed so that patients would be more likely to achieve a dose range associated with effectiveness for standard VNS, likely between 1.5 mA and 2.25 mA at 250–500 μs (25). Output current increases during these titration visits were not aided by fMRI.

### 2.4. Outcome measures and data collection

The primary effectiveness endpoint was the percent change in seizure frequency per month (over a 3-month period) compared to the seizure frequency per month (over the 3-month period) calculated at baseline, at 6 months post-implant, and 12 months post-implant. The primary safety endpoint was the occurrence of stimulation-related adverse events in the first 6 months after implant and in months 6 to 12 thereafter.

The study also assessed other secondary outcome measures related to seizure severity (Seizure Severity Questionnaire; SSQ), quality of life (Quality of Life in Epilepsy scales; QOLIE-31P, QOLIE-AD-48), medication load (prescribed daily dose/defined daily dose), and suicidality (Columbia Suicide Severity Rating Scale).

During the MRI scanning days, at the 2-week and 6-month visits, resting state fMRI was also collected from each patient as an exploratory outcome. This was collected at the beginning of the scanning day, shortly after the tolerability assessment but before any other MRI procedures were performed.

Study data were collected by site investigators or their designees and were entered into a custom-built, 21 CFR Part 11 compliant electronic data capture system managed by the study sponsor for subsequent analysis.

### 2.5. Statistical analysis methods

This clinical study was exploratory in nature. All the inferential statistics should be considered hypothesis-generating in nature and not confirmatory. Cohorts were not powered for the purpose of confirmatory statistical testing, and the population characteristics of each cohort are not expected to be suitable for a clinically meaningful comparison. Each cohort will be analyzed separately as soon as each cohort completes the relevant recruitment and subjects reach the expected follow-up threshold. Descriptive statistics (mean, standard deviation, median, mode, range, and confidence intervals, as appropriate) will be used to describe the population outcome of within-subject changes between baseline and each follow-up visit.

We plan for an intermediate analysis at the time of all subjects completing their 6-month follow-up visit. The final analysis was conducted when all subjects completed the study at the 12-month visit.

### 2.6. Withdrawal of consent and study exit

Subjects were permitted to withdraw their consent for the study at any time. Withdrawal of consent could be made through not signing a study-related form, through checking a box on that form indicating the subject's intention to withdraw their consent, or by emailing a representative of the sponsor directly if the subject was not actively completing study related forms.

Study investigators were also empowered to withdraw subjects from the study if they perceived a developing or active safety concern.

## 3. Discussion and design limitations

The study was designed to demonstrate the safety and potential efficacy of the investigational μVNS stimulation paradigm. In addition to this primary objective, an investigational fMRI protocol was employed to guide patients to an appropriate personalized dose of the therapy. The presence of two investigational variables in this study may increase the difficulty of assigning treatment effect sizes.

There were also risks to the study outcome driven by choices made in the design of the fMRI protocol. At the time of study design, the best choice of target ROI for standard vs. microburst was not clear; hence, a decision was made to use a thalamic ROI as the target measure of VNS response with adjustments based on the peak of BOLD responses. The selection was grounded in the data from the available literature including previous VNS neuroimaging studies (20–22, 26). It was also unclear at that time whether the peak is the best measure and whether, e.g., the volume of activated tissue in the thalamus or the volume of the overall activated brain should be used instead. The VNS cycle time in the scanner also created risks, as there is little available evidence in humans to confirm the validity of the 30 s off-time for washing out VNS effects in the central nervous system. Further, it was not feasible to analyze all available options for parameters; thus, it was possible to have missed an optimal parameter. Finally, randomization of treatment settings (intensity, or other μVNS parameters) was not conducted within each scan in order to reduce the risk of side effects that would impact the imaging procedure (e.g., a participant coughing during fMRI acquisition). Regarding the risk of bias driven by patient selection, there were no indications from the literature on whether the VNS treatment targets should be different between focal epilepsies and idiopathic/genetic generalized epilepsies.

Due to the complicating factor of the investigational fMRI titration paradigm, the investigators proposed a publication plan that specifically addresses subject outcomes during the titration phase separately from the longer-term outcomes. A pair of study outcomes manuscripts will be developed to address these matters in the future. In addition, one or more manuscripts focused on the potential mechanism of μVNS and its impact of resting state functional networks will be developed using the fMRI data.

## Ethics statement

The studies involving human participants were reviewed and approved by Northwestern University Biomedical IRB, Rush University Medical Center IRB, University of Utah IRB, Weill Cornell Medical College IRB, Western Institutional Review Board, Mayo Clinic Institutional Review Board, and Ethisch Comite UZ Gent. Written informed consent to participate in this study was provided by the participants' legal guardian/next of kin.

## Microburst Study Group

Danielle McDermott, University of Colorado and Denver Health; Mesha Gay Brown, University of Colorado School of Medicine; Lesley Kaye, University of Colorado School of Medicine; Michael Macken, Northwestern University; William O. Tatum, Mayo Clinic Florida; Cornelia Drees, Mayo Clinic Arizona; Selim R. Benbadis, University of South Florida; Zeenat Jaisani, University of Alabama at Birmingham; Muhammad Zafar, Duke University Hospital; Kristl Vonck, Ghent Unviersity Hospital; Rebecca O'Dwyer, Rush Epilepsy Center, Rush University Medical Center, Chicago, IL; Blake Newman, Univ of Utah School of Medicine; Pegah Afra, Weill-Cornell Medicine, University of Utah School of Medicine; Jane Allendorfer, University of Alabama at Birmingham (UAB) Department of Neurology; Jerzy P. Szaflarski, University of Alabama at Birmingham (UAB) Department of Neurology and the UAB Epilepsy Center, Birmingham, AL, USA; Ryan Verner, LivaNova PLC; Kathryn Nichol, LivaNova PLC; Charles Gordon, LivaNova PLC; Jason Begnaud, LivaNova PLC; Amy Keith, LivaNova PLC; Elhum Shamshiri, LivaNova PLC; Steffen Fetzer, LivaNova PLC; Giovanni Ranuzzi, LivaNova PLC; Gaia Giannicola, LivaNova PLC; Mei Jiang, LivaNova PLC; Wim Van Grunderbeek, LivaNova PLC; Irena Bellinski, Northwestern University; Elizabeth Cunningham, Northwestern University; Ann Mertens, Ghent Unviersity Hospital; Fiona Lynn, Rush Epilepsy Center, Rush University Medical Center, Chicago, IL; and Seyhmus Aydemir, Cornell.

## Author contributions

RV, JS, KV, and GG equally contributed to the initial draft of the manuscript. JA provided additional feedback during the draft revision process. The remainder of the Microburst Study Group offered critical review and feedback. All authors contributed to the article and approved the submitted version.
