# Supplementary material for: Modulation of the thalamus by microburst vagus nerve stimulation: a feasibility study protocol
Source: Front Neurol. 2023 Jun 13;14:1169161. doi: 10.3389/fneur.2023.1169161 (PMC10299807; doi:10.3389/fneur.2023.1169161)
Supplement: Supplementary file 1 [file Table_1.DOCX]

Supplementary Material

Modulation of the Thalamus by Microburst Vagus Nerve Stimulation: A Feasibility Study Protocol

Ryan Verner*, Jerzy P. Szaflarski, Jane B. Allendorfer, Kristl Vonck, Gaia Giannicola, on behalf of the Microburst Study Group

*** Correspondence:** Corresponding Author: ryan.verner@livanova.com

# Supplemental Table

Regulatory approval dates for the Clinical Investigation Plan and its amendments

| **Site** | **Name, Address of IRB/EC** | **CIP v A**  **15 JUN 2017** | | **CIP v B**  **31 AUG 2017** | | **CIP v C**  **28 AUG 2018** | |
| --- | --- | --- | --- | --- | --- | --- | --- |
|  |  | **Date of IRB/EC Approval** | **Date of Competent Authority (CA) Approval** | **Date of IRB/EC Approval** | **Date of Competent Authority (CA) Approval** | **Date of IRB/EC Approval** | **Date of Competent Authority (CA) Approval** |
| **Northwestern University** | **Name:**  Institutional Review Board  **Name/Address:**  Northwestern University  Biomedical IRB  750 N. Lake Shore Drive, Suite 700  Chicago, IL 60611 | NA | NA | 16 Nov 2017 | 01 Sep 2017 | 13 Nov 2018 | 31 Oct 2018 |
| **Rush University Medical**  **Center** | **Name:**  Institutional Review Board  **Name/Address:**  Rush University Medical Center  1653 W. Congress Parkway  Chicago, IL 60612 | NA | NA | 17 Jan 2018 | 01 Sep 2017 | 26 Nov 2018 | 31 Oct 2018 |
| **University of Utah** | **Name:**  Institutional Review Board  **Address:**  University of Utah  75 South 2000 East  Salt Lake City, Utah 84112 | NA | NA | 21 Aug 2018 | 01 Sep 2017 | 11 Jan 2019 | 31 Oct 2018 |
| **Weill Cornell Medical**  **College** | **Name:**  Institutional Review Board  **Address:**  1300 York Avenue, Box 89 New York, NY 10065 | NA | NA | 11 Dec 2018 | 01 Sep 2017 | 12 Mar 2019 | 31 Oct 2018 |
| **University of Alabama at**  **Birmingham** | **Name:**  Western Institutional Review Board  (WIRB)  **Address:**  1019 39th Avenue SE Suite 120  Puyallup, WA 98374-2115 | NA | NA | 04 Sep 2018 | 01 Sep 2017 | 30 Nov 2018 | 31 Oct 2018 |
| **University of Colorado**  **Denver** | **Name:**  Western Institutional Review Board  (WIRB)  **Address:**  1019 39th Avenue SE Suite 120  Puyallup, WA 98374-2115 | NA | NA | 18 Sep 2018 | 01 Sep 2017 | 30 Nov 2018 | 31 Oct 2018 |
| **Duke University** | **Name:**  Western Institutional Review Board  (WIRB)  **Address:**  1019 39th Avenue SE Suite 120  Puyallup, WA 98374-2115 | NA | NA | 25 Feb 2019 | 01 Sep 2017 | 25 Feb 2019 | 31 Oct 2018 |
| **Mayo Clinic Florida** | **Name:**  Mayo Clinic Institutional Review Board  **Address:**  201 Building, Room 4-60  200 First St. SW  Rochester, MN 55905 | NA | NA | NA | 01 Sep 2017 | 31 May 2019 | 31 Oct 2018 |
| **Universitair Ziekenhuis Gent** | **Name:**  Ethisch Comité UZ Gent  **Address:**  De Pintepark II, 2e verdieping  Corneel Heymanslaan 10  9000 Gent  Belgium | N/A | N/A | 25 Oct 2018 | 19 Mar 2019 | 06 Jun 2019 | 17 Jul 2019* |

*submission 17 Jul 2019, followed by tacit approval
